# Does transient serum phosphate or serum phosphate status cause chronic kidney disease-associated pruritus in peritoneal dialysis patients? A cross-sectional and group-based trajectory modeling study

**DOI:** 10.1080/0886022X.2025.2540557

**Published:** 2025-08-05

**Authors:** Zijun Zhou, Hanqing Zhang, Yunze Xing, Tao Zhang, Yaxuan Fang, Yueqi Zhang, Hanqi Yang, Junchen Li, Yintao Zhang, Bo Yang

**Affiliations:** ^a^ Department of Nephrology, First Teaching Hospital of Tianjin University of Traditional Chinese Medicine, Tianjin, China; ^b^ Department of Nephrology, National Clinical Research Center for Chinese Medicine Acupuncture and Moxibustion, Tianjin, China; ^c^ Graduate School, Tianjin University of Traditional Chinese Medicine, Tianjin, China

**Keywords:** Chronic kidney disease-associated pruritus, group-based trajectory modeling, peritoneal dialysis, persistent serum phosphate status, phosphorus-lowering therapy

## Abstract

**Background:**

Chronic kidney disease-associated pruritus (CKD-aP) is a common complication in peritoneal dialysis (PD) patients. Whether serum phosphate is associated with CKD-aP is controversial. We identified the serum phosphate trajectories of PD patients in the last 3 months and investigated the effects of serum phosphate persistence on CKD-aP.

**Methods:**

A total of 407 PD patients from the PD Center of the First Teaching Hospital of Tianjin University of Traditional Chinese Medicine were included. The 14-item UP-Dial was used to investigate the prevalence and clinical characteristics. PD patients were identified with different trajectories of serum phosphate by group-based trajectory modeling.

**Results:**

The prevalence of CKD-aP in PD patients was 51.8%. Among 407 PD patients, four distinct serum phosphate trajectories were identified. Both transient serum phosphate and serum phosphate status were associated with CKD-aP. Transient serum phosphate was associated with an increased risk of CKD-aP [OR (95%CI): 2.28 (1.30–4.02), 2.24 (1.22–4.10), 3.04 (1.66–5.58), 2.12 (1.21–3.70), respectively]. Compared with the persistent low-level group, the gradual increase group was not associated with an increased risk of CKD-aP [OR (95%CI): 1.59 (0.77–3.26)]. Both the gradual decrease group and the persistent high-level group were positively associated with an increased risk of CKD-aP [OR (95%CI): 2.18 (1.30–3.66), 3.19 (1.60–6.36), respectively].

**Conclusion:**

Our study confirmed the relationship between serum phosphate and CKD-aP within the past 3 months, to some extent explaining the controversy generated by previous studies. These results may help clinicians better understand the relationship between serum phosphate and CKD-aP and the importance of controlling serum phosphate.

## Introduction

Chronic kidney disease-associated pruritus (CKD-aP) is a common complication in peritoneal dialysis (PD) patients, with a prevalence ranging from 10% to 70% [1]. Meanwhile, CKD-aP is strongly associated with higher rates of hospitalization, technical failure, and all-cause mortality in PD patients, so focusing on CKD-aP is important for improving the quality of life and prognosis of long-term survival in PD patients [2,3].

CKD-aP is not given the attention it deserves in clinical practice. Firstly, the vast majority of nephrologists underestimate the prevalence of CKD-aP in clinical practice, with only 1% being able to accurately assess patients for pruritus [4,5]. Secondly, the risk factors associated with CKD-aP remain unelucidated, and most nephrologists attribute it to hyperphosphatemia, which is treated by lowering serum phosphate levels to alleviate the pruritus symptoms, but phosphorus-lowering therapy is minimally rewarding for many patients [6,7] and patients give up reporting their symptoms to doctors after receiving several ineffective treatments [6].

A growing number of studies have found that there does not appear to be a correlation between serum phosphate and CKD-aP [4,8–11]. Most of these studies are cross-sectional studies that only analyzed the relationship between serum phosphate and CKD-aP at the time of information collection, without investigating the persistent state of serum phosphate in patients. However, in real-world clinical practice, the levels of serum phosphate fluctuate continuously. The causes of CKD-aP may not be related to the values at a specific time point but rather the ongoing damage that ultimately leads to disease onset. This could also explain why phosphorus-lowering therapy is ineffective for some patients, as a brief treatment may not be enough to cure the ongoing damage.

We conducted a cross-sectional and group-based trajectory modeling study to (1) investigate the prevalence and clinical characteristics of CKD-aP in PD patients, (2) identify the serum phosphate trajectory in PD patients, (3) analyze the relationship between transient serum phosphate level or serum phosphate persistence status in the last 3 months and CKD-aP. These results may provide future directions for better prevention and treatment of CKD-aP in PD patients.

## Materials and methods

### Study design and population

The PD Center of the Department of Nephrology, the First Teaching Hospital of Tianjin University of Traditional Chinese Medicine, is one of the first PD demonstration centers in China. The center has over 800 registered PD patients, including all stages of PD patients (dialysis age from 0 months to more than 15 years), and has established individual PD files for each of these patients. This study selected PD patients who attended the center during the period from September 1, 2023, to June 1, 2024. The study is conducted under the Strengthening the Reporting of Observational Studies in Epidemiology (STROBE) guidelines [12]. Informed consent was obtained from all patients. Study approval was obtained from the Ethics Committee of the First Teaching Hospital of Tianjin University of Traditional Chinese Medicine (TYLL2023[Z]013).

This study was conducted in two parts (a cross-sectional analysis and a longitudinal analysis) to explore the relationship between serum phosphate and CKD-aP in a comprehensive and in-depth manner. First, in the cross-sectional analysis, we analyzed the serum phosphate measured at each time point within the past three months and CKD-aP for PD patients to initially explore whether there is an association between transient serum phosphate and CKD-aP. Second, in the longitudinal analysis, we determined the development trajectory of serum phosphate in PD patients over the past three months through the group-based trajectory modeling (GBTM) to deeply analyze the relationship between the short-term serum phosphate status and CKD-aP in PD patients.

We collected PD patients who undergo routine blood tests (serum phosphate, serum calcium, serum albumin) every month. The first measurement was recorded at this time. The first, second, and third months are the second, third, and fourth measurement time points, respectively. Consider the feasibility of clinical research, the observation period is three months, with a total of four-time points. At the fourth time point, the CKD-aP questionnaire assessment and clinical information collection are conducted.

Based on clinical practice experience and previous studies [13], we assumed the prevalence of CKD-aP in PD patients is about 50%, with a tolerance error of 5%. With an overall PD patient population of approximately 800 patients, a sample size of 260 cases was calculated as needed to complete the analysis. Considering that 15% of patients may be excluded according to the inclusion and exclusion criteria, the final sample size required for this study is at least 306 patients.

### Inclusion and exclusion criteria

Inclusion criteria were (1) regular PD > 3 months, (2) age > 18 years, (3) no symptoms of CKD-aP before 3 months by patients recall (To better explore the causal relationship between serum phosphorus and CKD-aP, only patients without CKD-aP at the time of collection data of the first time point were included to exclude the interference of reverse causality), and (4) cognitive function is normal, and able to cooperate with the information collection of this study. Exclusion criteria were (1) comorbidity with hemodialysis, (2) previous history of primary dermatosis (common diseases that cause skin itching, such as urticaria and eczema), (3) comorbidity with other diseases causing itchy skin, such as hepatic disease, (4) comorbidity with acute infections, 5) comorbidity with malignant tumors, 6) refusal to participate in the questionnaires [14].

### Assessment of CKD-aP

The diagnosis of CKD-aP requires the fulfillment of either of the following conditions: (1) the occurrence of itching several times in 1 day on 3 or more days within 2 weeks or less, with each itching symptom lasting more than a few minutes and troubling the patient, (2) regular pattern occurrence of pruritus symptoms within 6 months, but the frequency of itching can be less than the above situation [14,15].

The 14-item uremic pruritus in dialysis patients scale (14-item UP-Dial scale) was conducted for PD patients who met the diagnosis of CKD-aP to grade the severity of CKD-aP and to summarize the clinical features of CKD-aP. The questionnaire included 14 items covering three dimensions (pruritus symptoms, social psychological status, and sleep state), with each item scored from 0 to 4, for a total score of 0–56 points. The severity of CKD-aP was classified based on the score, mild [1–12], moderate [13–21], and severe (14, 16, 22–56). We used the Chinese version of the 14-item UP-Dial scale, this scale was translated and validated and has been confirmed to have high validity and reliability in the Chinese dialysis population [17].

### Data collection

After the 14-item UP-Dial scale survey, age, gender, body mass index (BMI), urea clearance index (Kt/V), time on dialysis, comorbidities (hypertension, diabetes mellitus, stroke), and medications (antihistamines, phosphate binders, calcitriol or vitamin D analogues, and calcimimetics) were collected. The serum phosphate (P), serum calcium (Ca), serum albumin (ALB), and albumin-adjusted serum calcium (adjusted Ca) of the patients first, second, third, and fourth measurement (0, 1, 2, 3 months) were collected. Since intact parathyroid hormone (iPTH) was generally measured once every three months, only the iPTH closest to the time of the questionnaire was collected. Age, sex, BMI, Kt/V, time on dialysis, iPTH, ALB, and adjusted Ca were included as covariates to exclude the effect of residual confounding.

Regarding the selection aspect of the baseline data, due to the relatively short three-month observation period, clinical characteristics (age, gender, BMI, Kt/V, and time on dialysis) were not significantly affected, and iPTH was measured only once within three months. Therefore, both longitudinal and cross-sectional analyses utilized these data uniformly collected at the endpoints. Serum phosphate, serum calcium, and serum albumin fluctuated during the three-month period, so the data from the first measurement were used as covariates in the longitudinal analysis and the data from each measurement were used as covariates in the cross-sectional analysis.

### Data analysis

Included PD patients were grouped by pruritus (yes/no) and baseline characteristics were presented. Normally distributed continuous variables were described by mean with SD and t-tests (or Welch’s t-test) were used. Skewed continuous variables were described by the median with IQR and the Mann–Whitney U-tests were used. Categorical variables were described by the counts with percentages and chi-square tests (or Yates’s correction for continuity/Fisher’s exact test) were used.

PD patients were identified with different development trajectories of serum phosphate by group-based trajectory modeling (GBTM). GBTM is a method for identifying individuals with similar trajectories of change over time. It is a data-derived statistical algorithm, rather than relying on the subjective classification of the researcher, and has more advantages in the analysis of longitudinal data [18,19]. Regarding the selection of GBTM type: Firstly, in combination with the fluctuation of serum phosphate levels in clinical practice, we excluded the linear model. Secondly, due to the short observation period, the proportion of patients with repeated fluctuations in serum phosphate levels over a three-month period was relatively small, so the cubic model was excluded. Finally, it was determined that the quadratic model was used for the analysis. The specific trajectory was also finalized by combining clinical practice experience and the parameters of the quadratic GBTM. The model parameters conform to the following requirements as much as possible: (1) The average posterior probability (AvePP) for each group exceeds 0.7; (2) The proportion of each category is at least 5%; (3) The AIC and BIC approaches 0; (4) The relative entropy exceeds 0.8; (5) The OCC is greater than 5.

Associations were analyzed by logistic regression and confounding factors were excluded by 3 models. Model 1 did not include any covariate (crude model). Model 2 was adjusted for age, biologic sex, BMI, and Kt/V, time on dialysis. Model 3 was adjusted for sex, gender, BMI, Kt/V, time on dialysis, iPTH, adjusted Ca, and ALB.

To verify the robustness of the results, we conducted several sensitivity analyses. First, we reanalyzed the data after using propensity score matching (PSM) to eliminate the differences between the two groups (The specific matching criteria are detailed in the footnote of Supplementary data, Table S4.). Second, we classified serum phosphate according to 1.78 mmol/L (5.5 mg/dl) to explore the relationship between therapeutic target values and CKD-aP in PD patients in the clinical practice. Third, we divided CKD-aP severity according to the 14-item UP-Dial scale to explore the relationship between serum phosphate and CKD-aP severity. Finally, we grouped age, sex, BMI, Kt/V, and time on dialysis to explore the association between different subgroups.

All statistical calculations were performed using R version 4.4.1 and SPSS version 29.0 (IBM, Armonk, NY, USA). In all analyses we allowed for a type 1 error of 5%, with all hypotheses being two-sided.

## Results

### Prevalence and clinical features

**Figure 1.**
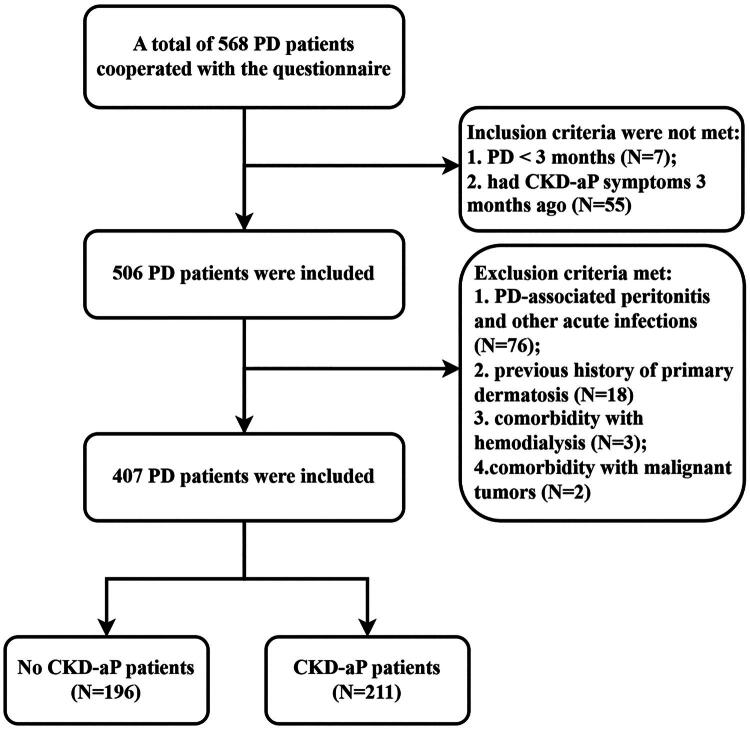
Flow chart of the study patients. We screened the patients who participated in the questionnaire according to the inclusion and exclusion criteria and finally identified 407 patients who met the criteria.

**Figure 2.**
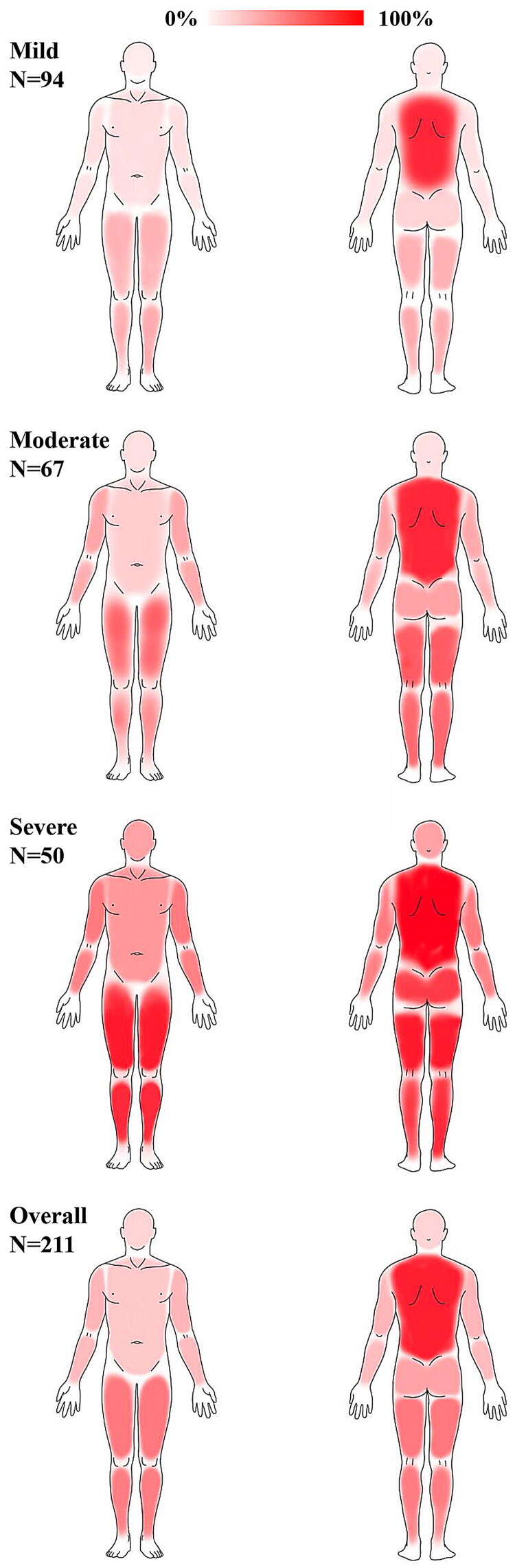
Distribution of pruritus area in different degrees of CKD-aP patients. The depth of red indicates the percentage of patients with pruritus in this area in the whole group, and the redder the area, the more patients with pruritus. The pruritus area was mainly concentrated in the back and waist, thigh, and lower leg.

A total of 407 PD patients met the inclusion criteria, including 196 patients (48.2%) without CKD-aP and 211 patients (51.8%) with varying degrees of CKD-aP (mild: 94, 23.1%; moderate: 67, 16.5%; severe: 50, 12.3%), the flow chart is shown in Figure 1. The distributions of CKD-aP were mostly symmetrical, mainly concentrated in the back and waist (158, 74.9%), thigh (104, 49.3%), and lower leg (101, 47.9%), and spread to the whole body with the aggravation of itching (Figure 2, Supplementary data, Table S1).

### Identified the serum phosphate trajectory

**Figure 3.**
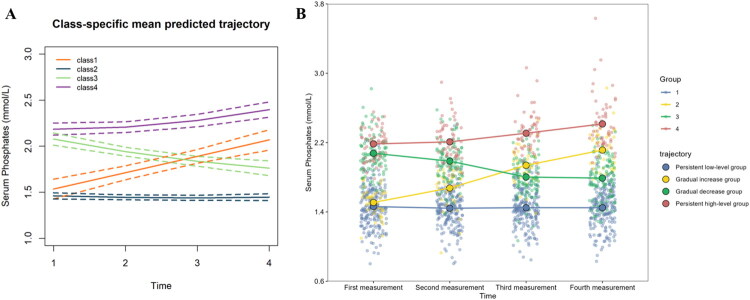
The serum phosphate trajectories in peritoneal dialysis patients. We selected 4 types of trajectory classification: persistent low-level group, gradual increase group, gradual decrease group, and persistent high-level group. In Figure 3A: Class 1, gradual increase group; class 2, persistent low-level group; class 3, gradual decrease group; class 4, persistent high-level group. In Figure 3B: Scatter marks the value of each measurement in the population for that trajectory. It can be seen that the trajectory identified by GBTM (Figure 3A) agrees with the trajectory of the actual distribution (Figure 3B). The first measurement was 0 month; the second measurement was the first month; the third measurement was the second month; the fourth measurement was the third month.

We performed GBTM analysis of 4 times serum phosphate measurements from 407 patients, pre-establishing 2 to 5 models. Combined with the model parameters (Supplementary data, Table S2 and Figure S1) and clinical interpretability, we selected 4 types of trajectory classification (Figures 3(A,B)): persistent low-level group (210, 51.6%), gradual increase group (41, 10.1%), gradual decrease group (101, 24.8%), and persistent high-level group (55, 13.5%). The baseline characteristics of patients of different trajectories are shown in Supplementary data, Table S3.

### Baseline characteristics

Compared with patients without CKD-aP, patients with CKD-aP had a significant higher proportion of males (*p* = 0.001), a lower Kt/V (*p* < 0.001), more usage of antihistamines (*p* = 0.027), higher serum phosphate (*p* = 0.005, *p* = 0.015, *p* = 0.001, *p* = 0.010, each measurement), and a lower ALB (*p* = 0.003, *p* < 0.001, *p* = 0.018, *p* < 0.001, each measurement), the difference is statistically significant. No significant differences were found in age, BMI, time on dialysis, comorbidities, medications (phosphate binders, calcitriol or vitamin D analogues, and calcimimetics), iPTH, Ca, and adjusted Ca. Patients with CKD-aP had a more gradual decrease group (29.4% vs 19.9%) and persistent high-level group (18.0% vs 8.7%) and a less persistent low-level group (43.1% vs 60.7%) than patients without CKD-aP (*p* < 0.001) (Table 1).

**Table 1.** Characteristics of peritoneal dialysis patients, stratified by presence of CKD-aP (yes/no) at baseline.

Parameters	Total patients *N* = 407	No CKD-aP *N* = 196	CKD-aP *N* = 211	*P*-value
Clinical characteristic				
Age (years), mean (SD)	57.42 ± 12.07	56.44 ± 13.19	58.33 ± 10.89	0.118
Sex (male), N (%)	228 (56.0)	97 (49.5)	131 (62.1)	0.011
BMI (kg/m^2^], mean (SD)	24.68 ± 4.26	24.32 ± 4.07	25.03 ± 4.41	0.093
Kt/V, mean (SD)	1.75 ± 0.37	1.84 ± 0.35	1.66 ± 0.37	<0.001
Time on dialysis (month), median (IQR)	39.0 (16.5, 60.0)	39.0 (17.0, 51.0)	39.0 (15.0, 63.0)	0.366
Comorbidity history (yes), N (%)				
Hypertension	402 (98.8)	192 (98.0)	210 (99.5)	0.325
Diabetes mellitus	188 (46.2)	86 (43.9)	102 (48.3)	0.367
Stroke	69 (17.0)	29 (14.8)	40 (19.0)	0.264
Medications (yes), N (%)				
Antihistamines	12 (2.9)	2 (1.0)	10 (4.7)	0.027
Phosphate binders	298 (73.2)	146 (74.5)	152 (72.0)	0.577
Calcitriol/Vitamin D analogues	177 (43.5)	89 (45.4)	88(47.1)	0.452
Calcimimetics	60 (14.7)	26 (131.3)	34 (16.1)	0.418
Laboratory value				
iPTH (pg/ml), median (IQR)	385.60 (244.35, 564.60)	419.35 (252.88, 602.70)	365.90 (238.40, 506.95)	0.067
First measurement*				
P (mmol/L), mean (SD)	1.72 ± 0.39	1.66 ± 0.35	1.77 ± 0.41	0.005
Ca (mmol/L), mean (SD)	2.13 ± 0.18	2.14 ± 0.17	2.13 ± 0.18	0.613
adjusted Ca^#^ (mmol/L), mean (SD)	2.25 ± 0.18	2.24 ± 0.16	2.26 ± 0.19	0.158
ALB (g/L), mean (SD)	35.27 ± 4.67	35.98 ± 4.57	34.62 ± 4.68	0.003
Second measurement*				
P (mmol/L), mean (SD)	1.70 ± 0.37	1.66 ± 0.35	1.75 ± 0.39	0.015
Ca (mmol/L), mean (SD)	2.13 ± 0.17	2.13 ± 0.17	2.12 ± 0.17	0.283
adjusted Ca^#^(mmol/L), mean (SD)	2.25 ± 0.17	2.24 ± 0.17	2.26 ± 0.17	0.172
ALB (g/L), mean (SD)	35.06 ± 4.77	35.92 ± 4.63	34.26 ± 4.78	<0.001
Third measurement*				
P (mmol/L), mean (SD)	1.70 ± 0.37	1.64 ± 0.33	1.76 ± 0.40	0.001
Ca (mmol/L), mean (SD)	2.14 ± 0.19	2.14 ± 0.16	2.13 ± 0.21	0.528
adjusted Ca^#^ (mmol/L), mean (SD)	2.26 ± 0.19	2.25 ± 0.17	2.27 ± 0.20	0.378
ALB (g/L), mean (SD)	35.07 ± 4.84	35.66 ± 4.80	34.53 ± 4.78	0.018
Fourth measurement*				
P (mmol/L), mean (SD)	1.73 ± 0.42	1.68 ± 0.39	1.78 ± 0.44	0.010
Ca (mmol/L), mean (SD)	2.13 ± 0.18	2.12 ± 0.18	2.13 ± 0.18	0.795
adjusted Ca^#^(mmol/L), mean (SD)	2.25 ± 0.21	2.20 ± 0.19	2.30 ± 0.22	<0.001
ALB (g/L), mean (SD)	35.01 ± 5.14	36.98 ± 4.44	33.19 ± 5.08	<0.001
P trajectories, N (%)				<0.001
Persistent low-level group	210 (51.6)	119 (60.7)	91 (43.1)	
Gradual increase group	41 (10.1)	21 (10.7)	20 (9.5)	
Gradual decrease group	101 (24.8)	39 (19.9)	62 (29.4)	
Persistent high-level group	55 (13.5)	17 (8.7)	38 (18.0)	

Abbreviations: IQR, interquartile range; SD, standard deviation; CKD-aP, chronic kidney disease-associated pruritus; BMI, body mass index; Kt/V, urea clearance index; P, serum phosphate; Ca, Serum calcium; iPTH, intact parathyroid hormone; ALB, Serum albumin.

*: The first measurement was 0 month; the second measurement was the first month; the third measurement was the second month; the fourth measurement was the third month.

#: Albumin-adjusted serum calcium.

### Association between serum phosphate and CKD-aP

Both transient serum phosphate and serum phosphate status were significantly associated with CKD-aP and this association persisted after adjustment for covariates (Table 2). Serum phosphate measured at 4 different time points was significantly associated with an increased risk of CKD-aP [OR (95%CI): 2.28 (1.30–4.02), 2.24 (1.22–4.10), 3.04 (1.66–5.58), 2.12 (1.21–3.70), respectively]. In the results of the serum phosphate state analysis, compared with the persistent low-level group, the gradual increase group was not associated with an increased risk of CKD-aP [OR (95%CI): 1.59 (0.77–3.26)]. In contrast, both the gradual decrease group and the persistent high-level group were positively associated with an increased risk of CKD-aP [OR (95%CI): 2.18 (1.30–3.66), 3.19 (1.60–6.36), respectively].

**Table 2.** Associations between serum phosphate and CKD-aP.

Parameters	Model 1 [OR (95% CI)]	*P*-value	Model 2 [OR (95% CI)]	*P*-value	Model 3 [OR (95% CI)]	*P*-value
Transient P						
First measurement P	2.08 (1.24–3.49)	0.005	2.02 (1.16–3.50)	0.013	2.28 (1.30–4.02)	0.004
Second measurement P	1.93 (1.13–3.28)	0.016	1.77 (1.00–3.14)	0.051	2.24 (1.22–4.10)	0.009
Third measurement P	2.41 (1.39–4.17)	0.002	2.63 (1.46–4.73)	0.001	3.04 (1.66–5.58)	<0.001
Fourth measurement P	1.86 (1.15–3.00)	0.011	1.99 (1.19–3.32)	0.008	2.12 (1.21–3.70)	0.008
P trajectories						
Persistent low-level group	Reference	–	Reference	–	Reference	–
Gradual increase group	1.25 (0.64–2.44)	0.521	1.48 (0.73–3.00)	0.277	1.59 (0.77–3.26)	0.211
Gradual decrease group	2.08 (1.28–3.38)	0.003	2.00 (1.21–3.30)	0.007	2.18 (1.30–3.66)	0.003
Persistent high-level group	2.92 (1.55–5.51)	<0.001	2.82 (1.44–5.52)	0.002	3.19 (1.60–6.36)	<0.001

Abbreviations: OR, odds ratio; CI, confidence interval; P, serum phosphate.

The first measurement was 0 month; the second measurement was the first month; the third measurement was the second month; the fourth measurement was the third month.

Model 1 did not include any covariate (crude model); Model 2 was adjusted for age, sex, BMI, and Kt/V, time on dialysis; Model 3 was adjusted for age, sex, BMI, Kt/V, time on dialysis, iPTH, adjusted Ca, ALB.

To verify the robustness of the results, we performed a 1:1 PSM and reanalysis to rule out baseline imbalances between the two groups. There were no differences in baseline characteristics after PSM (Supplementary data, Table S4). After adjustment for covariates, the results for PSM were similar to those of the overall population (Table 3). Serum phosphate measured at 4 different time points was significantly associated with an increased risk of CKD-aP [OR (95%CI): 2.24 (1.07–4.66), 2.42 (1.14–5.14), 4.38 (1.98–9.69), 3.01 (1.33–6.79), respectively]. Compared with the persistent low-level group, the gradual increase group was not associated with an increased risk of CKD-aP [OR (95%CI): 1.90 (0.67–5.33)]. Both the gradual decrease group and the persistent high-level group were positively associated with an increased risk of CKD-aP [OR (95%CI): 2.10 (1.11–3.97), 4.39 (1.72–11.17), respectively].

**Table 3.** Associations between serum phosphate and CKD-aP, after propensity score matching.

Parameters	Model 1 [OR (95% CI)]	*P*-value	Model 2 [OR (95% CI)]	*P*-value	Model 3 [OR (95% CI)]	*P*-value
Transient P						
First measurement P	2.02 (1.11–3.66)	0.021	1.98 (1.01–3.89)	0.048	2.24 (1.07–4.66)	0.032
Second measurement P	1.75 (0.96–3.19)	0.066	1.73 (0.88–3.39)	0.112	2.42 (1.14–5.14)	0.022
Third measurement P	2.50 (1.33–4.68)	0.004	3.11 (1.51–6.42)	0.002	4.38 (1.98–9.69)	<0.001
Fourth measurement P	2.10 (1.15–3.82)	0.015	2.69 (1.35–5.34)	0.005	3.01 (1.33–6.79)	0.008
P trajectories						
Persistent low-level group	Reference	–	Reference	–	Reference	–
Gradual increase group	1.07 (0.45–2.56)	0.875	1.52 (0.59–3.90)	0.382	1.90 (0.67–5.33)	0.225
Gradual decrease group	1.86 (1.07–3.21)	0.027	1.98 (1.09–3.59)	0.025	2.10 (1.11–3.97)	0.023
Persistent high-level group	2.94 (1.39–6.20)	0.005	3.67 (1.54–8.74)	0.003	4.39 (1.72–11.17)	0.002

Abbreviations: OR, odds ratio; CI, confidence interval; P, serum phosphate.

The first measurement was 0 month; the second measurement was the first month; the third measurement was the second month; the fourth measurement was the third month.

Model 1 did not include any covariate (crude model); Model 2 was adjusted for age, sex, BMI, and Kt/V, time on dialysis; Model 3 was adjusted for age, sex, BMI, Kt/V, time on dialysis, iPTH, adjusted Ca, ALB.

We also categorized 4 times serum phosphorus measured according to therapeutic target values (1.78 mmol/L) to verify the robustness of the results (Supplementary data, Table S5). The results of the post-classification reanalysis were similar to the previous results after adjusting for covariates (Table 4). Serum phosphate above 1.78 mmol/L was positively associated with an increased risk of CKD-aP compared with acceptable hyperphosphatemia in clinical practice, but this association was not found for serum phosphate measured at the time of the questionnaire survey [OR (95%CI): 2.46 (1.58–3.93), 2.33 (1.48–3.67), 2.23 (1.43–3.49), 1.46 (0.92–2.31), respectively].

**Table 4.** Associations between serum phosphate (stratified by 1.78 mmol/L) and CKD-aP.

Parameters	Model 1 [OR (95% CI)]	*P*-value	Model 2 [OR (95% CI)]	*P*-value	Model 3 [OR (95% CI)]	*P*-value
First measurement						
*p* ≤ 1.78 mmol/L	Reference	–	Reference	–	Reference	–
*p* > 1.78 mmol/L	2.29 (1.52–3.44)	<0.001	2.24 (1.46–3.45)	<0.001	2.46 (1.58–3.93)	<0.001
Second measurement						
*p* ≤ 1.78 mmol/L	Reference	–	Reference	–	Reference	–
*p* > 1.78 mmol/L	2.15 (1.43–3.23)	<0.001	1.94 (1.26–2.98)	0.003	2.33 (1.48–3.67)	<0.001
Third measurement						
*p* ≤ 1.78 mmol/L	Reference	–	Reference	–	Reference	–
*p* > 1.78 mmol/L	1.938 (1.29–2.92)	0.001	2.05 (1.33–3.18)	0.001	2.23 (1.43–3.49)	<0.001
Fourth measurement						
*p* ≤ 1.78 mmol/L	Reference	–	Reference	–	Reference	–
*p* > 1.78 mmol/L	1.46 (0.98–2.18)	0.065	1.46 (0.96–2.24)	0.081	1.46 (0.92–2.31)	0.110

Abbreviations: OR, odds ratio; CI, confidence interval; P, serum phosphate. The first measurement was 0 month; the second measurement was the first month; the third measurement was the second month; the fourth measurement was the third month.

Model 1 did not include any covariate (crude model); Model 2 was adjusted for age, sex, BMI, and Kt/V, time on dialysis; Model 3 was adjusted for age, sex, BMI, Kt/V, time on dialysis, iPTH, adjusted Ca, ALB.

### Associations between serum phosphate and degree of CKD-aP

To further investigate the relationship between serum phosphate and the degree of CKD-aP, we classified the CKD-aP patients by the 14-item UP-Dial scale scores. There were differences in age, Kt/V, and ALB among the 3 groups (Supplementary data, Table S6). Serum phosphate measured 2 times in the recent past was positively associated with the degree of CKD-aP [OR (95%CI): 2.54 (1.24–5.23), 2.36 (1.24–4.49), respectively], but two times in the previous period was not associated with the degree of CKD-aP [OR (95%CI): 1.30 (0.66–2.55), 2.02 (0.95–4.31), respectively]. The gradual decrease group was associated with the degree of CKD-aP compared with the persistent low-level group [OR (95%CI): 1.15 (0.60–2.20)]. Both the gradual increase group and the persistent high-level group were associated with the degree of CKD-aP [OR (95%CI): 2.59 (1.01–6.65), 2.84 (1.25–6.42), respectively] (Table 5).

**Table 5.** Associations between serum phosphate and degree of CKD-aP (stratified by mild/moderate/severe).

Parameters	Model 1 [OR (95% CI)]	*P*-value	Model 2 [OR (95% CI)]	*P*-value	Model 3 [OR (95% CI)]	*P*-value
Transient P						
First measurement P	1.03 (0.56–1.90)	0.918	1.21 (0.62–2.36)	0.570	1.30 (0.66–2.55)	0.445
Second measurement P	1.37 (0.71–2.62)	0.346	1.79 (0.85–3.75)	0.123	2.02 (0.95–4.31)	0.069
Third measurement P	1.55 (0.82–2.93)	0.179	2.40 (1.17–4.89)	0.016	2.54 (1.24–5.23)	0.011
Fourth measurement P	1.81 (1.02–3.22)	0.041	2.49 (1.33–4.68)	0.004	2.36 (1.24–4.49)	0.009
P trajectories						
Persistent low-level group	Reference	–	Reference	–	Reference	–
Gradual increase group	2.04 (0.83–5.01)	0.118	2.54 (1.00–6.50)	0.051	2.59 (1.01–6.65)	0.048
Gradual decrease group	1.02 (0.55–1.87)	0.954	1.07 (0.56–2.04)	0.835	1.15 (0.60–2.20)	0.678
Persistent high-level group	2.30 (1.13–4.66)	0.021	3.30 (1.51–7.24)	0.003	3.31 (1.50–7.32)	0.003

Abbreviations: OR, odds ratio; CI, confidence interval; P, serum phosphate.

The first measurement was 0 month; the second measurement was the first month; the third measurement was the second month; the fourth measurement was the third month.

Model 1 did not include any covariate (crude model); Model 2 was adjusted for age, sex, BMI, and Kt/V, time on dialysis; Model 3 was adjusted for age, sex, BMI, Kt/V, time on dialysis, iPTH, adjusted Ca, ALB.

### Subgroup analyses

To verify the robustness of the results, we performed a series of subgroup analyses (Table 6). In the analysis of the relationship between transient serum phosphate and CKD-aP, we observed the subgroups with age < 65 years, BMI ≥ 24.0 kg/m^2^, Kt/*V* < 1.7, and time on dialysis ≥ 60 months had a positive association between four times serum phosphate measurements and an increased risk of CKD-aP. In males, the recent serum phosphate and CKD-aP were more closely related, while in females, CKD-aP was more related to the earlier serum phosphate. In the analysis of the relationship between serum phosphate status and CKD-aP, the subgroups with age < 65 years, male, and time on dialysis < 60 months had a positive association between the persistent high-level group and the risk of CKD-aP. In the subgroup with age ≥ 65 years, the gradual decrease group had a positive association with the risk of CKD-aP. In the subgroup with female sex, BMI ≥ 24.0 kg/m^2^, Kt/*V* < 1.7, time on dialysis ≥ 60 months, both the persistent high-level group and the gradual decrease group were positively associated with the risk of CKD.

**Table 6.** Associations between serum phosphate and CKD-aP in different subgroups (stratified by age, gender, BMI, Kt/V, and time on dialysis).

Parameters	OR (95% CI)	*P*-value	Parameters	OR (95% CI)	*P*-value	P for interaction
Age < 65 years (*N* = 266)			Age ≥ 65 years (*N* = 141)			
Transient P			Transient P			
First measurement P	2.04 (1.03–4.06)	0.041	First measurement P	2.85 (0.96–8.44)	0.059	0.364
Second measurement P	2.27 (1.09–4.70)	0.028	Second measurement P	1.73 (0.57–5.23)	0.332	0.831
Third measurement P	3.39 (1.62–7.08)	0.001	Third measurement P	1.99 (0.67–5.90)	0.212	0.665
Fourth measurement P	2.32 (1.16–4.65)	0.017	Fourth measurement P	1.71 (0.61–4.79)	0.310	0.793
P trajectories			P trajectories			
Persistent low-level group	Reference	–	Persistent low-level group	Reference	–	
Gradual increase group	2.04 (0.87–4.80)	0.102	Gradual increase group	0.60 (0.14–2.68)	0.506	
Gradual decrease group	1.75 (0.93–3.32)	0.085	Gradual decrease group	3.84 (1.38–10.70)	0.010	
Persistent high-level group	3.18 (1.38–7.32)	0.006	Persistent high-level group	2.54 (0.71–9.08)	0.151	
Gender (male) (*N* = 228)			Gender (female) (*N* = 179)			
Transient P			Transient P			
First measurement P	1.95 (0.92–4.14)	0.081	First measurement P	3.23 (1.28–8.12)	0.013	0.720
Second measurement P	2.03 (0.89–4.60)	0.091	Second measurement P	3.15 (1.23–8.04)	0.016	0.972
Third measurement P	4.52 (1.11–5.71)	0.027	Third measurement P	4.20 (1.64–10.75)	0.003	0.751
Fourth measurement P	2.36 (1.10–5.07)	0.028	Fourth measurement P	2.02 (0.85–4.79)	0.112	0.617
P trajectories			P trajectories			
Persistent low-level group	Reference	–	Persistent low-level group	Reference	–	
Gradual increase group	1.84 (0.72–4.71)	0.205	Gradual increase group	1.49 (0.45–4.96)	0.512	
Gradual decrease group	1.83 (0.93–3.57)	0.080	Gradual decrease group	3.39 (1.44–7.98)	0.005	
Persistent high-level group	2.90 (1.15–7.34)	0.025	Persistent high-level group	3.84 (1.31–11.24)	0.014	
BMI < 24.0 kg/m^2^ (*N* = 191)			BMI ≥ 24.0 kg/m^2^ (*N* = 216)			
Transient P			Transient P			
First measurement P	2.29 (0.94–5.63)	0.070	First measurement P	2.31 (1.06–5.04)	0.035	0.291
Second measurement P	1.81 (0.75–4.37)	0.186	Second measurement P	2.63 (1.10–6.29)	0.029	0.097
Third measurement P	1.95 (0.75–5.04)	0.170	Third measurement P	3.37 (1.46–7.78)	0.004	0.091
Fourth measurement P	1.35 (0.54–3.35)	0.516	Fourth measurement P	2.68 (1.29–5.58)	0.008	0.126
P trajectories			P trajectories			
Persistent low-level group	Reference	–	Persistent low-level group	Reference	–	
Gradual increase group	1.15 (0.41–3.27)	0.789	Gradual increase group	2.01 (0.67–6.03)	0.211	
Gradual decrease group	2.23 (0.99–5.01)	0.052	Gradual decrease group	2.09 (1.03–4.21)	0.041	
Persistent high-level group	2.34 (0.71–7.67)	0.162	Persistent high-level group	3.31 (1.41–7.78)	0.006	
Kt/*V* < 1.7 (*N* = 227)			Kt/*V* ≥ 1.7 (*N* = 180)			
Transient P			Transient P			
First measurement P	3.14 (1.42–6.95)	0.005	First measurement P	1.42 (0.59–3.41)	0.437	0.132
Second measurement P	3.51 (1.45–8.51)	0.005	Second measurement P	1.25 (0.52–3.05)	0.618	0.104
Third measurement P	5.91 (2.25–15.49)	<0.001	Third measurement P	1.55 (0.67–3.58)	0.306	0.045
Fourth measurement P	3.27 (1.44–7.44)	0.005	Fourth measurement P	1.29 (0.56–2.97)	0.547	0.121
P trajectories			P trajectories			
Persistent low-level group	Reference	–	Persistent low-level group	Reference	–	
Gradual increase group	1.33 (0.47–3.78)	0.592	Gradual increase group	1.20 (0.43—3.32)	0.727	
Gradual decrease group	2.12 (1.06–4.21)	0.033	Gradual decrease group	2.13 (0.91–4.97)	0.080	
Persistent high-level group	7.42 (2.36–23.30)	<0.001	Persistent high-level group	1.76 (0.63–4.88)	0.278	
Time on dialysis < 60 months (*N* = 305)			Time on dialysis ≥ 60 months (*N* = 102)			
Transient P			Transient P			
First measurement P	1.47 (0.77–2.80)	0.242	First measurement P	12.24 (2.66–56.35)	0.001	0.003
Second measurement P	1.49 (0.76–2.92)	0.246	Second measurement P	13.88 (2.34–82.26)	0.004	0.001
Third measurement P	2.38 (1.20–4.75)	0.013	Third measurement P	9.11 (1.95–42.47)	0.005	0.125
Fourth measurement P	1.66 (0.87–3.18)	0.125	Fourth measurement P	7.20 (1.74–29.83)	0.007	0.116
P trajectories			P trajectories			
Persistent low-level group	Reference	–	Persistent low-level group	Reference	–	
Gradual increase group	1.20 (0.55–2.64)	0.652	Gradual increase group	6.50 (0.57–74.71)	0.133	
Gradual decrease group	1.48 (0.80–2.75)	0.212	Gradual decrease group	6.87 (1.83–25.80)	0.004	
Persistent high-level group	2.76 (1.25–6.11)	0.012	Persistent high-level group	11.96 (1.34–106.61)	0.026	

Abbreviations: OR, odds ratio; CI, confidence interval; P, serum phosphate.

The first measurement was 0 month; the second measurement was the first month; the third measurement was the second month; the fourth measurement was the third month.

Adjusted for age, sex, BMI, Kt/V, time on dialysis, iPTH, adjusted Ca, and ALB (excluding grouped variables).

## Discussion

In this cross-sectional study of 407 PD patients, we observed that the prevalence of varying degrees of CKD-aP in PD patients was 51.8%, and the pruritus area was mainly concentrated in the back and waist, thigh, and lower leg. Both transient serum phosphate and serum phosphate status were strongly associated with the risk of CKD-aP. Our findings may largely explain the inconsistent conclusions of previous studies on the relationship between serum phosphate and CKD-aP.

In this study, serum phosphate was positively associated with the risk of CKD-aP at each of the 4 times measured within the past 3 months. The results of the multiple sensitivity analyses were similar to the primary results, confirming the robustness of our results. Serum phosphate measured 2 times in the recent past was positively associated with the degree of CKD-aP. Indeed, serum phosphate has been a classical risk factor for CKD-aP [1,20–23], so most medical directors consider serum phosphate control as the most important therapeutic option in the clinical management of CKD-aP [4,21]. This is consistent with our findings regarding transient serum phosphate. However, some studies have found no association between serum phosphate and CKD-aP after correction for confounders [4,8–11,24]. These conclusions are not contradictory, as most studies only concentrate on transient serum phosphate and do not focus on the effect of persistent serum phosphate status. It is not advisable to determine a causal relationship based solely on transient serum phosphate, because it is easily affected by many factors, such as it may simply be that the patient did not control their diet for a few days before the test [25].

Therefore, in this study, we focused on the relationship between the trajectory of serum phosphate and CKD-aP in PD patients within the past 3 months. We observed that compared to the persistent low-level group, the gradual increase group had no higher risk of CKD-aP, whereas both the persistent high-level group and the gradual decrease group were associated with an increased risk of CKD-aP. In subsequent sensitivity analyses, the persistent high-level group and the gradual decrease group remained associated with a higher risk of CKD-aP. There was also a strong association between the severity of CKD-aP and the persistent high-level group. Interestingly, in our study, although the gradual increasing group was not associated with the risk of CKD-aP, it was related to the severity of CKD-aP. This largely explains the previous controversy. First, our study confirmed that persistent high serum phosphate levels are indeed closely related to CKD-aP. Second, we observed that a gradually decreasing serum phosphate trajectory was positively associated with CKD-aP, but a gradually increasing serum phosphate trajectory was not related to CKD-aP. For a common cross-sectional study, the different proportions of these 2 groups may directly affect the conclusion. No dialysis center can manage dialysis patients in the same way, which may be the main reason for the controversy.

Our findings also explain, in part, why most medical directors still choose serum phosphate control as the most important therapeutic option for the treatment of CKD-aP even though there are no published interventional studies showing effectiveness [4]. Patients with persistently high levels of serum phosphate are more likely to suffer from CKD-aP than those with persistently low levels. A gradual increase in serum phosphate may also lead to a worsening of CKD-aP symptoms in patients. Although the gradual decrease group is also associated with a higher risk of CKD-aP, this may be because chronic damage caused by the previous high-level state has already occurred. Improvement in symptoms often has a certain lag, which may also be why some patients feel that phosphorus-lowering treatment is ineffective. Regardless, long-term strengthening of serum phosphate control and maintaining it at a long-term stable low level is crucial for improving CKD-aP.

CKD-aP is caused by a combination of pathogenic factors. A cohort study that included 108,679 dialysis patients found that patients older than 65 years were more likely to have CKD-aP [2]. Kt/V has also been shown to be negatively associated with the risk of CKD-aP [3,20,26], and a previous study demonstrated no association between serum phosphate and CKD-aP in patients with Kt/*V* > 1.7 [8]. To better control for the influence of some recognized influencing factors of CKD-aP on the conclusion, we also conducted subgroup analyses to explore which populations have the strongest association between CKD-aP and serum phosphate. Our study found that high serum phosphate levels were associated with an increased risk of CKD-aP in patients with age < 65 years, male, BMI ≥ 24.0 kg/m^2^, Kt/*V* < 1.7, and time on dialysis ≥ 60 months, and the gradual decrease group had a positive association with the risk of CKD-aP in patients with age ≥ 65 years. In patients with female, BMI ≥ 24.0 kg/m^2^, Kt/*V* < 1.7, time on dialysis ≥ 60 months, both the persistent high-level group and the gradual decrease group were positively associated with the risk of CKD. There is also controversy regarding the effect of gender on CKD-aP [22,27], and our results showed that CKD-aP was associated with transient serum phosphate in male patients, whereas with serum phosphorus status in female patients.

Our study has certain limitations. Firstly, we only analyzed serum phosphorus trajectories within the last 3 months and did not observe longer trajectories, and perhaps patients in the consistently lowering group did not have an increased risk of CKD-aP after a period of lowering. It is also possible that serum phosphate is constantly fluctuating in the clinic, which may require more measurement points for identification. Secondly, our study did not collect the dietary information of the patients before measurement, which may affect the measurement results. In addition, during the observation period, we only measured iPTH once, which might have affected our comprehensive assessment of the association between CKD-aP and serum calcium, serum phosphate, and iPTH. Our study also lacks direct evidence of serum phosphate deposition damage, which may be unfavorable for further explanation of the pathogenesis of serum phosphate and CKD-aP. Finally, limited by the cross-sectional study and questionnaire survey, our results should be viewed with caution. The assessment of whether patients had CKD-aP was carried out based on recollections, therefore could not avoid recall bias.

In conclusion, our study identified the serum phosphorus trajectories of PD patients. It confirmed the relationship between serum phosphate and CKD-aP within the past 3 months, to some extent explaining the controversy generated by previous studies. These results may help clinicians better understand the relationship between serum phosphate and CKD-aP and the importance of controlling serum phosphate.

## Supplementary Material

Supplementary_material_Clean.docx

## Data Availability

Data can be requested by contacting the corresponding author of the article.
