# Supplementary material for: Does transient serum phosphate or serum phosphate status cause chronic kidney disease-associated pruritus in peritoneal dialysis patients? A cross-sectional and group-based trajectory modeling study
Source: Ren Fail. 2025 Aug 5;47(1):2540557. doi: 10.1080/0886022X.2025.2540557 (PMC12329824; doi:10.1080/0886022X.2025.2540557)
Supplement: Supplementary_material_Clean.docx [file IRNF_A_2540557_SM6948.docx]

Table of contents Supplemental Tables

Table S1. Distribution of pruritus area in different degrees of CKD-aP patients.

Table S2. The selection process of GBTM

Table S3. Characteristics of peritoneal dialysis patients, stratified by serum phosphate trajectories.

Table S4. Characteristics of peritoneal dialysis patients, before and after propensity score matching

Table S5. Distribution of serum phosphate (stratified by 1.78mmol/L) in CKD-aP patients.

Table S6. Characteristics of peritoneal dialysis patients, stratified by degree of CKD-aP (mild/moderate/severe) at baseline

Figure S1. Identified trajectories of serum phosphate.

Table S1. Distribution of pruritus area in different degrees of CKD-aP patients.

| Distribution of pruritic | Mild CKD-aP  N=94 | Moderate CKD-aP  N=67 | Severe CKD-aP  N=50 | Overall CKD-aP  N=211 |
| --- | --- | --- | --- | --- |
| Head and face | 8(8.5%) | 8(11.9%) | 17(34.0%) | 33(15.6%) |
| Forearms | 9(9.6%) | 21(31.3%) | 23(46.0%) | 53(25.1%) |
| Upper arm | 11(11.7%) | 22(32.8%) | 24(48.0%) | 57(27.0%) |
| Chest and Abdomen | 12(12.8%) | 13(19.4%) | 19(38.0%) | 44(20.9%) |
| Back and Waist | 69(73.4%) | 49(73.1%) | 40(80.0%) | 158(74.9%) |
| Buttocks | 15(16.0%) | 23(34.3%) | 34(68.0%) | 72(34.1%) |
| Thigh | 27(28.7%) | 39(58.2%) | 38(76.0%) | 104(49.3%) |
| Lower leg | 29(30.9%) | 36(53.7%) | 36(72.0%) | 101(47.9%) |

Abbreviations: CKD-aP, chronic kidney disease-associated pruritus.

Table S2. The selection process of GBTM

| P trajectories | Proportions per class (%) | AvePP | OCC | Entropy | BIC | AIC | LL |
| --- | --- | --- | --- | --- | --- | --- | --- |
| 2-group solution | 59.95/40.05 | 0.97/0.94 | 23.91/25.87 | 0.86 | 937.50 | 905.43 | -444.72 |
| 3-group solution | 53.81/35.14/11.06 | 0.96/0.91/0.86 | 21.33/17.61/51.12 | 0.85 | 809.10 | 760.99 | -368.50 |
| 4-group solution | 10.07/51.60/24.82/13.51 | 0.86/0.95/0.85/0.93 | 44.22/17.88/18.32/80.38 | 0.84 | 746.96 | 682.83 | -325.41 |
| 5-group solution | 4.18/49.39/9.34/23.59/13.51 | 0.90/0.91/0.85/0.88/0.91 | 132.21/11.45/47.98/24.24/67.96 | 0.83 | 724.45 | 644.28 | -302.14 |

Abbreviations: GBTM, group-based trajectory modeling; P, serum phosphate; AvePP, average posterior probability; OCC, odds of correct classification; BIC, Bayesian information criterion; AIC, Akaike information criterion; LL, Log-likelihood.

Table S3. Characteristics of peritoneal dialysis patients, stratified by serum phosphate trajectories.

| Parameters | Total patients  N=407 | Persistent low-level group  N=210 | Gradual increase group  N=41 | Gradual decrease group  N=101 | Persistent high-level group  N=55 | *P*-value |
| --- | --- | --- | --- | --- | --- | --- |
| Clinical characteristic |  |  |  |  |  |  |
| Age (years), mean (SD) | 57.42±12.07 | 58.64±12.00 | 56.63±12.87 | 56.65±11.42 | 54.76±12.66 | 0.145 |
| Gender (male), N (%) | 228 (56.0) | 114 (54.3) | 23 (56.1) | 59 (58.4) | 32 (58.2) | 0.897 |
| BMI (kg/m2), mean (SD) | 24.68±4.26 | 24.01±3.52 | 24.46±4.46 | 25.00±4.03 | 26.84±6.08 | <0.001 |
| Kt/V, mean (SD) | 1.75±0.37 | 1.77±0.39 | 1.88±0.39 | 1.69±0.31 | 1.68±0.33 | 0.016 |
| Time on dialysis (month),  median (IQR) | 39.0  (16.5, 60.0) | 36.5  (15.0, 57.0) | 33  (15.0, 50.0) | 39  (24.0, 63.0) | 41  (27.0, 57.0) | 0.120 |
| Comorbidity history (yes), N (%) |  |  |  |  |  |  |
| Hypertension | 402 (98.8) | 207 (98.6) | 40 (97.6) | 100 (99.0) | 55 (100.0) | 0.735 |
| Diabetes mellitus | 188 (46.2) | 94 (44.8) | 23 (56.1) | 43 (42.6) | 28 (50.9) | 0.421 |
| Stroke | 69 (17.0) | 37 (17.6) | 11 (26.8) | 12 (11.9) | 9 (16.4) | 0.190 |
| Medications (yes), N (%) |  |  |  |  |  |  |
| Antihistamines | 12 (2.9) | 2 (1.0) | 2 (4.9) | 4 (4.0) | 4 (7.3) | 0.025 |
| Phosphate binders | 298 (73.2) | 136 (64.8) | 32 (78.0) | 87 (86.1) | 43 (78.2) | <0.001 |
| Calcitriol/Vitamin D analogues | 177 (43.5) | 90 (42.9) | 12 (29.3) | 52 (51.5) | 23 (41.8) | 0.107 |
| Calcimimetics | 60 (14.7) | 22 (10.5) | 7 (17.1) | 21 (20.8) | 10 (18.2) | 0.083 |
| Laboratory value |  |  |  |  |  |  |
| iPTH (pg/ml), median (IQR) | 385.60  (244.35, 564.60) | 367.25  (231.20, 576.00) | 318.00  (253.30, 551.40) | 386.30  (237.70, 548.70) | 435.00  (313.90, 435.00) | 0.250 |
| First measurement^*^ |  |  |  |  |  |  |
| P (mmol/L), mean (SD) | 1.72±0.39 | 1.46±0.24 | 1.51±0.17 | 2.08±0.22 | 2.18±0.18 | <0.001 |
| Ca (mmol/L), mean (SD) | 2.13±0.18 | 2.13±0.19 | 2.16±0.21 | 2.13±0.15 | 2.12±0.17 | 0.768 |
| adjusted Ca^#^ (mmol/L),  mean (SD) | 2.25±0.18 | 2.27±0.19 | 2.25±0.19 | 2.23±0.15 | 2.24±0.18 | 0.312 |
| ALB (g/L), mean (SD) | 35.27±4.67 | 34.71±4.83 | 36.21±4.42 | 36.02±4.40 | 35.37±4.55 | 0.061 |
| Second measurement^*^ |  |  |  |  |  |  |
| P (mmol/L), mean (SD) | 1.70±0.37 | 1.44±0.22 | 1.68±0.21 | 1.99±0.22 | 2.21±0.24 | <0.001 |
| Ca (mmol/L), mean (SD) | 2.13±0.17 | 2.14±0.18 | 2.13±0.15 | 2.12±0.15 | 2.08±0.16 | 0.105 |
| adjusted Ca^#^ (mmol/L),  mean (SD) | 2.25±0.17 | 2.28±0.18 | 2.25±0.15 | 2.22±0.16 | 2.20±0.17 | 0.003 |
| ALB (g/L), mean (SD) | 35.06±4.77 | 34.53±5.11 | 35.40±4.52 | 35.98±4.43 | 35.14±3.99 | 0.086 |
| Third measurement^*^ |  |  |  |  |  |  |
| P (mmol/L), mean (SD) | 1.70±0.37 | 1.45±0.21 | 1.93±0.21 | 1.80±0.22 | 2.31±0.23 | <0.001 |
| Ca (mmol/L), mean (SD) | 2.14±0.19 | 2.13±0.19 | 2.13±0.14 | 2.16±0.16 | 2.14±0.24 | 0.771 |
| adjusted Ca^#^ (mmol/L),  mean (SD) | 2.26±0.19 | 2.27±0.19 | 2.24±0.16 | 2.26±0.16 | 2.25±0.24 | 0.701 |
| ALB (g/L), mean (SD) | 35.07±4.84 | 34.48±5.08 | 35.44±4.57 | 36.03±4.44 | 35.32±4.60 | 0.057 |
| Fourth measurement^*^ |  |  |  |  |  |  |
| P (mmol/L), mean (SD) | 1.73±0.42 | 1.45±0.22 | 2.11±0.23 | 1.79±0.24 | 2.42±0.30 | <0.001 |
| Ca (mmol/L), mean (SD) | 2.13±0.18 | 2.12±0.18 | 2.12±0.17 | 2.16±0.17 | 2.11±0.21 | 0.143 |
| adjusted Ca^#^ (mmol/L),  mean (SD) | 2.25±0.21 | 2.23±0.20 | 2.24±0.20 | 2.30±0.24 | 2.25±0.24 | 0.081 |
| ALB (g/L), mean (SD) | 35.01±5.14 | 35.35±5.10 | 35.46±5.10 | 34.57±5.36 | 34.24±4.92 | 0.364 |

Abbreviations: IQR, interquartile range; SD, standard deviation; BMI, body mass index; Kt/V, urea clearance index; P, serum phosphate; Ca, Serum calcium; iPTH, intact parathyroid hormone; ALB, Serum albumin.

*: The first measurement was 0 month; the second measurement was the first month; the third measurement was the second month; the fourth measurement was the third month.

#: Albumin-adjusted serum calcium.

Table S4. Characteristics of peritoneal dialysis patients, before and after propensity score matching

| Parameters | Before propensity score matching | | | |  | After propensity score matching^&^ | | | |
| --- | --- | --- | --- | --- | --- | --- | --- | --- | --- |
|  | No CKD-aP  N=196 | CKD-aP  N=211 | *P*-value | SMD |  | No CKD-aP  N=164 | CKD-aP  N=164 | *P*-value | SMD |
| Clinical characteristic |  |  |  |  |  |  |  |  |  |
| Age (years), mean (SD) | 56.44±13.19 | 58.33±10.89 | 0.118 | 0.156 |  | 56.86±12.43 | 57.60±10.99 | 0.566 | 0.063 |
| Gender (male), N (%) | 97 (49.5) | 131 (62.1) | 0.011 | 0.256 |  | 91 (55.5) | 95 (57.9) | 0.656 | 0.049 |
| BMI (kg/m^2^), mean (SD) | 24.32±4.07 | 25.03±4.41 | 0.093 | 0.168 |  | 24.75±4.03 | 24.79±4.29 | 0.929 | 0.010 |
| Kt/V, mean (SD) | 1.84±0.35 | 1.66±0.37 | <0.001 | 0.489 |  | 1.76±0.29 | 1.71±0.32 | 0.123 | 0.171 |
| Time on dialysis (month),  median (IQR) | 39.0  (17.0, 51.0) | 39.0  (15.0, 63.0) | 0.366 | 0.052 |  | 39.0  (17.0, 51.0) | 38.00  (15.0, 61.5) | 0.826 | 0.012 |
| Comorbidity history (yes), N (%) |  |  |  |  |  |  |  |  |  |
| Hypertension | 192 (98.0) | 210 (99.5) | 0.325 | 0.141 |  | 163 (99.4) | 163 (99.4) | 1.000 | <0.001 |
| Diabetes mellitus | 86 (43.9) | 102 (48.3) | 0.367 | 0.090 |  | 73 (44.5) | 77 (47.0) | 0.658 | 0.049 |
| Stroke | 29 (14.8) | 40 (19.0) | 0.264 | 0.111 |  | 26 (15.9) | 24 (14.6) | 0.759 | 0.034 |
| Medications (yes), N (%) |  |  |  |  |  |  |  |  |  |
| Antihistamines | 2 (1.0) | 10 (4.7) | 0.027 | 0.224 |  | 2 (1.2) | 0 (0.0) | 0.156 | 0.157 |
| Phosphate binders | 146 (74.5) | 152 (72.0) | 0.577 | 0.055 |  | 120 (73.2) | 117 (71.3) | 0.711 | 0.041 |
| Calcitriol/Vitamin D analogues | 89 (45.4) | 88(47.1) | 0.452 | 0.075 |  | 72 (43.9) | 70 (42.7) | 0.824 | 0.025 |
| Calcimimetics | 26 (131.3) | 34 (16.1) | 0.418 | 0.081 |  | 20 (12.2) | 23 (14.0) | 0.624 | 0.054 |
| Laboratory value |  |  |  |  |  |  |  |  |  |
| iPTH (pg/ml), median (IQR) | 419.35  (252.88, 602.70) | 365.90  (238.40, 506.95) | 0.067 | 0.150 |  | 424.50  (254.60, 612.43) | 353.70  (235.32, 505.17) | 0.030 | 0.184 |
| First measurement^*^ |  |  |  |  |  |  |  |  |  |
| P (mmol/L), mean (SD) | 1.66±0.35 | 1.77±0.41 | 0.005 | 0.281 |  | 1.66±0.35 | 1.76±0.42 | 0.021 | 0.257 |
| Ca (mmol/L), mean (SD) | 2.14±0.17 | 2.13±0.18 | 0.613 | 0.050 |  | 2.14±0.16 | 2.12±0.18 | 0.250 | 0.127 |
| adjusted Ca^#^ (mmol/L),  mean (SD) | 2.24±0.16 | 2.26±0.19 | 0.158 | 0.141 |  | 2.24±0.16 | 2.26±0.19 | 0.305 | 0.114 |
| ALB (g/L), mean (SD) | 35.98±4.57 | 34.62±4.68 | 0.003 | 0.293 |  | 36.09±4.47 | 34.40±4.65 | 0.001 | 0.371 |
| Second measurement^*^ |  |  |  |  |  |  |  |  |  |
| P (mmol/L), mean (SD) | 1.66±0.35 | 1.75±0.39 | 0.015 | 0.243 |  | 1.66±0.35 | 1.73±0.40 | 0.068 | 0.202 |
| Ca (mmol/L), mean (SD) | 2.13±0.17 | 2.12±0.17 | 0.283 | 0.107 |  | 2.13±0.17 | 2.11±0.17 | 0.162 | 0.155 |
| adjusted Ca^#^ (mmol/L),  mean (SD) | 2.24±0.17 | 2.26±0.17 | 0.172 | 0.136 |  | 2.23±0.16 | 2.26±0.17 | 0.219 | 0.136 |
| ALB (g/L), mean (SD) | 35.92±4.63 | 34.26±4.78 | <0.001 | 0.354 |  | 36.03±4.64 | 34.05±4.76 | <0.001 | 0.421 |
| Third measurement^*^ |  |  |  |  |  |  |  |  |  |
| P (mmol/L), mean (SD) | 1.64±0.33 | 1.76±0.40 | 0.001 | 0.320 |  | 1.63±0.33 | 1.75±0.41 | 0.004 | 0.32 |
| Ca (mmol/L), mean (SD) | 2.14±0.16 | 2.13±0.21 | 0.528 | 0.063 |  | 2.14±0.16 | 2.12±0.21 | 0.488 | 0.077 |
| adjusted Ca^#^ (mmol/L),  mean (SD) | 2.25±0.17 | 2.27±0.20 | 0.378 | 0.088 |  | 2.25±0.16 | 2.26±0.21 | 0.361 | 0.101 |
| ALB (g/L), mean (SD) | 35.66±4.80 | 34.53±4.78 | 0.018 | 0.236 |  | 35.72±4.95 | 34.38±4.74 | 0.012 | 0.278 |
| Fourth measurement^*^ |  |  |  |  |  |  |  |  |  |
| P (mmol/L), mean (SD) | 1.68±0.39 | 1.78±0.44 | 0.010 | 0.256 |  | 1.67±0.38 | 1.77±0.43 | 0.019 | 0.260 |
| Ca (mmol/L), mean (SD) | 2.12±0.18 | 2.13±0.18 | 0.795 | 0.026 |  | 2.12±0.18 | 2.13±0.18 | 0.536 | 0.068 |
| adjusted Ca^#^ (mmol/L),  mean (SD) | 2.20±0.19 | 2.30±0.22 | <0.001 | 0.477 |  | 2.20±0.19 | 2.30±0.23 | <0.001 | 0.506 |
| ALB (g/L), mean (SD) | 36.98±4.44 | 33.19±5.08 | <0.001 | 0.794 |  | 36.96±4.53 | 33.18±5.24 | <0.001 | 0.772 |
| P trajectories, N (%) |  |  | <0.001 | 0.412 |  |  |  | 0.018 | 0.356 |
| Persistent low-level group | 119 (60.7) | 91 (43.1) |  |  |  | 99 (60.4) | 76 (46.3) |  |  |
| Gradual increase group | 21 (10.7) | 20 (9.5) |  |  |  | 16 (9.8) | 13 (7.9) |  |  |
| Gradual decrease group | 39 (19.9) | 62 (29.4) |  |  |  | 35 (21.3) | 46 (28.1) |  |  |
| Persistent high-level group | 17 (8.7) | 38 (18.0) |  |  |  | 14 (8.5) | 29 (17.7) |  |  |

Abbreviations: IQR, interquartile range; SD, standard deviation; SMD, standardized mean differences; CKD-aP, chronic kidney disease-associated pruritus; BMI, body mass index; Kt/V, urea clearance index; P, serum phosphate; Ca, Serum calcium; iPTH, intact parathyroid hormone; ALB, Serum albumin.

&: We performed propensity score matching (PSM) for all variables except laboratory tests using the nearest neighbor caliper matching method (1:1 without replacement). The propensity scores were calculated through logistic regression [Variables include: Clinical characteristic (age, gender, BMI, and Kt/V, time on dialysis), Comorbidity history (hypertension, diabetes mellitus, stroke), and Medications: antihistamines, phosphate binders, calcitriol or vitamin D analogues, and calcimimetics)], and the matching was conducted in descending order of the propensity scores with a caliper value of 0.2.

*: The first measurement was 0 month; the second measurement was the first month; the third measurement was the second month; the fourth measurement was the third month.

#: Albumin-adjusted serum calcium.

Table S5. Distribution of serum phosphate (stratified by 1.78mmol/L) in CKD-aP patients.

| Parameters^*^ | Total patients N=407 | No CKD-aP N=196 | CKD-aP N=211 | *P*-value |
| --- | --- | --- | --- | --- |
| First measurement, N (%) |  |  |  | <0.001 |
| P ≤1.78mmol/L | 241 (59.2) | 136 (69.4) | 105 (49.8) |  |
| P >1.78mmol/L | 166 (40.8) | 60 (30.6) | 106 (50.2) |  |
| Second measurement, N (%) |  |  |  | <0.001 |
| P ≤1.78mmol/L | 242 (59.5) | 135 (68.9) | 107 (50.7) |  |
| P >1.78mmol/L | 165 (40.5) | 61 (31.1) | 104 (49.3) |  |
| Third measurement, N (%) |  |  |  | 0.001 |
| P ≤1.78mmol/L | 252 (61.9) | 137 (69.9) | 115 (54.5) |  |
| P >1.78mmol/L | 155 (38.1) | 59 (30.1) | 96 (45.5) |  |
| Fourth measurement, N (%) |  |  |  | 0.064 |
| P ≤1.78mmol/L | 249 (61.2) | 129 (65.8) | 120 (56.9) |  |
| P >1.78mmol/L | 158 (38.8) | 67 (34.2) | 91 (43.1) |  |

Abbreviations: P, serum phosphate.

*: The first measurement was 0 month; the second measurement was the first month; the third measurement was the second month; the fourth measurement was the third month.

Table S6. Characteristics of peritoneal dialysis patients, stratified by degree of CKD-aP (mild/moderate/severe) at baseline

| Parameters | Total CKD-aP patients  N=211 | Mild CKD-aP  N=94 | Moderate CKD-aP  N=67 | Severe CKD-aP  N=50 | *P*-value |
| --- | --- | --- | --- | --- | --- |
| Clinical characteristic |  |  |  |  |  |
| Age (years), mean (SD) | 58.33±10.89 | 55.04±12.34 | 60.76±9.28 | 61.26±8.10 | <0.001 |
| Gender (male), N (%) | 131 (62.1) | 48 (51.1) | 47(70.1) | 36 (72.0) | 0.012 |
| BMI, mean (SD) | 25.03±4.41 | 25.23±5.31 | 25.47±3.95 | 24.05±2.75 | 0.191 |
| Kt/V, mean (SD) | 1.66±0.37 | 1.75±0.35 | 1.60±0.40 | 1.58±0.33 | 0.007 |
| Time on dialysis (month),  median (IQR) | 39.0 (15.0, 63.0) | 39.0 (15.0, 63.0) | 39.0 (18.0, 58.0) | 47.0 (18.0, 67.0) | 0.553 |
| Comorbidity history (yes), N (%) |  |  |  |  |  |
| Hypertension | 210 (99.5) | 93 (98.9) | 67 (100.0) | 50 (100.0) | 1.000 |
| Diabetes mellitus | 102 (48.3) | 40 (42.6) | 35 (52.2) | 27 (54.0) | 0.315 |
| Stroke | 40 (19.0) | 17 (18.1) | 12 (17.9) | 11 (22.0) | 0.821 |
| Medications (yes), N (%) |  |  |  |  |  |
| Antihistamines | 10 (4.7) | 1 (1.1) | 3 (4.5) | 6 (12.0) | 0.012 |
| Phosphate binders | 152 (72.0) | 67 (71.3) | 51 (76.1) | 34 (68.0) | 0.611 |
| Calcitriol/Vitamin D analogues | 88 (47.1) | 44 (46.8) | 26 (38.8) | 18 (36.0) | 0.385 |
| Calcimimetics | 34 (16.1) | 13 (13.8) | 10 (14.9) | 11 (22.0) | 0.424 |
| Laboratory value |  |  |  |  |  |
| iPTH (pg/ml), median (IQR) | 365.90 (238.40, 506.95) | 350.75 (237.1, 456.3) | 385.60 (257.25, 570.25) | 367.25 (228.40, 519.40) | 0.727 |
| First measurement^*^ |  |  |  |  |  |
| P (mmol/L), mean (SD) | 1.77±0.41 | 1.77±0.38 | 1.75±0.44 | 1.79±0.43 | 0.890 |
| Ca (mmol/L), mean (SD) | 2.13±0.18 | 2.14±0.16 | 2.11±0.19 | 2.13±0.22 | 0.630 |
| adjusted Ca^#^ (mmol/L),  mean (SD) | 2.26±0.19 | 2.26±0.17 | 2.26±0.19 | 2.28±0.24 | 0.825 |
| ALB (g/L), mean (SD) | 34.62±4.68 | 35.26±4.67 | 34.22±4.70 | 33.96±4.62 | 0.196 |
| Second measurement^*^ |  |  |  |  |  |
| P (mmol/L), mean (SD) | 1.75±0.39 | 1.71±0.39 | 1.81±0.36 | 1.74±0.41 | 0.223 |
| Ca (mmol/L), mean (SD) | 2.12±0.17 | 2.14±0.16 | 2.08±0.17 | 2.11±0.18 | 0.095 |
| adjusted Ca^#^ (mmol/L),  mean (SD) | 2.26±0.17 | 2.26±0.15 | 2.25±0.17 | 2.28±0.20 | 0.748 |
| ALB (g/L), mean (SD) | 34.26±4.78 | 35.34±4.47 | 33.34±4.78 | 33.44±5.02 | 0.012 |
| Third measurement^*^ |  |  |  |  |  |
| P (mmol/L), mean (SD) | 1.76±0.40 | 1.71±0.40 | 1.82±0.39 | 1.77±0.41 | 0.209 |
| Ca (mmol/L), mean (SD) | 2.13±0.21 | 2.16±0.20 | 2.13±0.22 | 2.09±0.19 | 0.225 |
| adjusted Ca^#^ (mmol/L),  mean (SD) | 2.27±0.20 | 2.27±0.20 | 2.28±0.22 | 2.26±0.19 | 0.911 |
| ALB (g/L), mean (SD) | 34.53±4.78 | 35.48±4.50 | 34.10±4.78 | 33.31±5.22 | 0.025 |
| Fourth measurement^*^ |  |  |  |  |  |
| P (mmol/L), mean (SD) | 1.78±0.44 | 1.71±0.41 | 1.82±0.44 | 1.86±0.50 | 0.124 |
| Ca (mmol/L), mean (SD) | 2.13±0.18 | 2.15±0.17 | 2.12±0.20 | 2.09±0.18 | 0.154 |
| adjusted Ca^#^ (mmol/L),  mean (SD) | 2.30±0.22 | 2.28±0.21 | 2.32±0.25 | 2.30±0.21 | 0.504 |
| ALB (g/L), mean (SD) | 33.19±5.08 | 34.87±4.95 | 31.79±5.14 | 31.91±4.35 | <0.001 |
| P trajectories, N (%) |  |  |  |  | 0.152 |
| Persistent low-level group | 91 (43.1) | 47 (50.0) | 24 (35.8) | 20 (40.0) |  |
| Gradual increase group | 20 (9.5) | 6 (6.4) | 8 (11.9) | 6 (12.0) |  |
| Gradual decrease group | 62 (29.4) | 30 (31.9) | 21 (31.3) | 11 (22.0) |  |
| Persistent high-level group | 38 (18.0) | 11 (11.7) | 14 (20.9) | 13 (26.0) |  |

Abbreviations: IQR, interquartile range; SD, standard deviation; CKD-aP, chronic kidney disease-associated pruritus; BMI, body mass index; Kt/V, urea clearance index; P, serum phosphate; Ca, Serum calcium; iPTH, intact parathyroid hormone; ALB, Serum albumin.

*: The first measurement was 0 month; the second measurement was the first month; the third measurement was the second month; the fourth measurement was the third month.

#: Albumin-adjusted serum calcium.


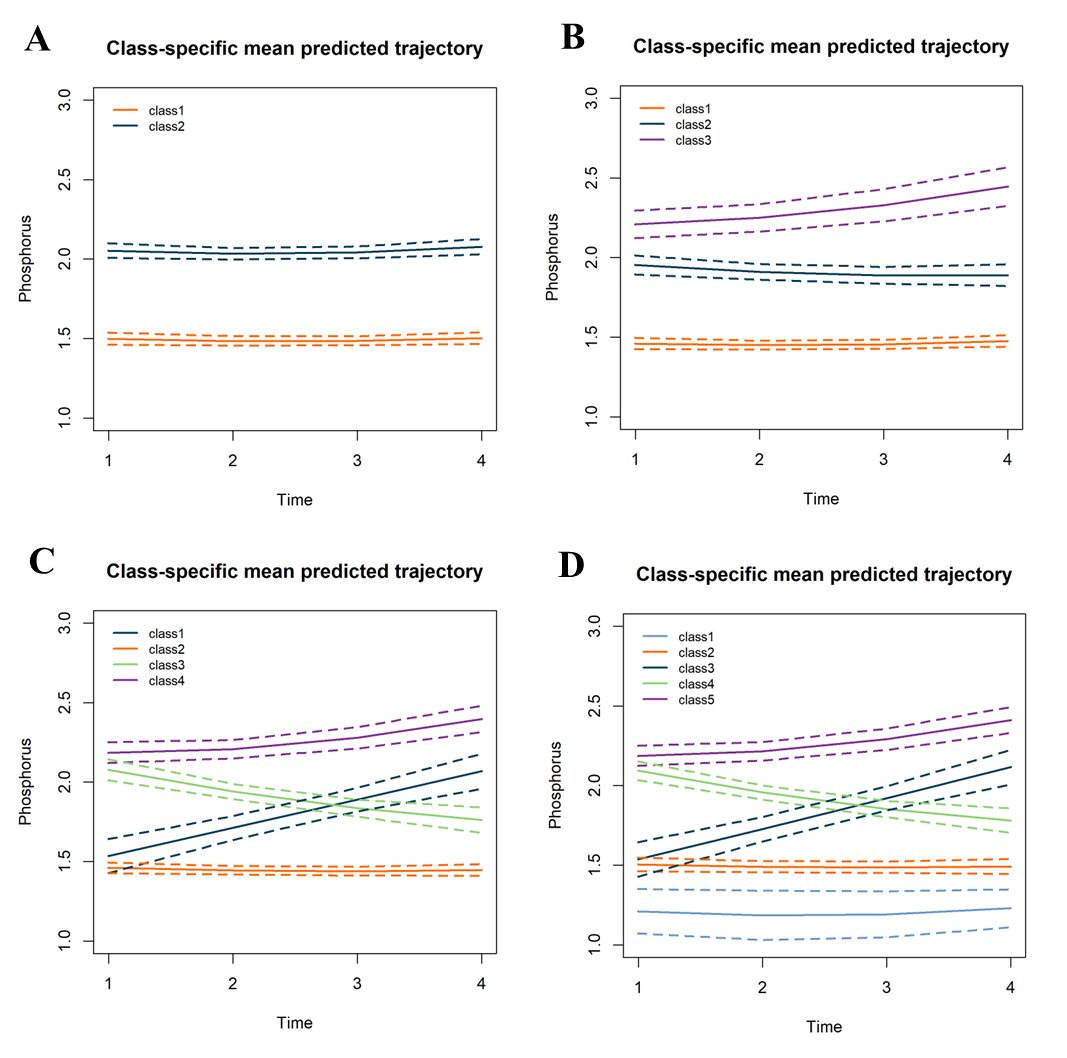


Figure S1. Identified trajectories of serum phosphate. We performed GBTM analysis of 4 times serum phosphorus measurements from 407 patients, pre-establishing 2 to 5 models. Combined with the model parameters and clinical interpretability, we selected 4 types of trajectory classification.
